# Machine learning-based prediction models for home discharge in patients with COVID-19: Development and evaluation using electronic health records

**DOI:** 10.1371/journal.pone.0292888

**Published:** 2023-10-20

**Authors:** Ruben D. Zapata, Shu Huang, Earl Morris, Chang Wang, Christopher Harle, Tanja Magoc, Mamoun Mardini, Tyler Loftus, François Modave

**Affiliations:** 1 Department of Health Outcomes and Biomedical Informatics, University of Florida College of Medicine, Gainesville, FL, United States of America; 2 Department of Pharmaceutical Outcomes and Policy, University of Florida College of Pharmacy, Gainesville, FL, United States of America; 3 Clinical and Translational Science Institute, University of Florida, Gainesville, FL, United States of America; 4 Department of Surgery, University of Florida College of Medicine, Gainesville, FL, United States of America; 5 Department of Anesthesiology, University of Florida College of Medicine, Gainesville, FL, United States of America; Kyung Hee University School of Medicine, REPUBLIC OF KOREA

## Abstract

**Objective:**

This study aimed to develop and validate predictive models using electronic health records (EHR) data to determine whether hospitalized COVID-19-positive patients would be admitted to alternative medical care or discharged home.

**Methods:**

We conducted a retrospective cohort study using deidentified data from the University of Florida Health Integrated Data Repository. The study included 1,578 adult patients (≥18 years) who tested positive for COVID-19 while hospitalized, comprising 960 (60.8%) female patients with a mean (SD) age of 51.86 (18.49) years and 618 (39.2%) male patients with a mean (SD) age of 54.35 (18.48) years. Machine learning (ML) model training involved cross-validation to assess their performance in predicting patient disposition.

**Results:**

We developed and validated six supervised ML-based prediction models (logistic regression, Gaussian Naïve Bayes, k-nearest neighbors, decision trees, random forest, and support vector machine classifier) to predict patient discharge status. The models were evaluated based on the area under the receiver operating characteristic curve (ROC-AUC), precision, accuracy, F1 score, and Brier score. The random forest classifier exhibited the highest performance, achieving an accuracy of 0.84 and an AUC of 0.72. Logistic regression (accuracy: 0.85, AUC: 0.71), k-nearest neighbor (accuracy: 0.84, AUC: 0.63), decision tree (accuracy: 0.84, AUC: 0.61), Gaussian Naïve Bayes (accuracy: 0.84, AUC: 0.66), and support vector machine classifier (accuracy: 0.84, AUC: 0.67) also demonstrated valuable predictive capabilities.

**Significance:**

This study’s findings are crucial for efficiently allocating healthcare resources during pandemics like COVID-19. By harnessing ML techniques and EHR data, we can create predictive tools to identify patients at greater risk of severe symptoms based on their medical histories. The models developed here serve as a foundation for expanding the toolkit available to healthcare professionals and organizations. Additionally, explainable ML methods, such as Shapley Additive Explanations, aid in uncovering underlying data features that inform healthcare decision-making processes.

## Introduction

Severe acute respiratory syndrome coronavirus 2 (SARS-CoV-2) is a virus that was identified in 2019. A highly contagious disease, SARS-CoV-2 causes the COVID-19 disease and has been declared a public health emergency by the World Health Organization (WHO) [1]. Since 2020, COVID-19 has greatly burdened the global health system. As of December 2021, nearly 60 million cases and over 800,000 deaths were reported in the United States alone [2]. Patients with severe COVID-19 require an average of 13 days’ worth of respiratory support [3] in the emergency room. As such, hospital systems are unable to allocate resources accordingly when the number of patients rapidly increases, requiring new methods to be developed for identifying patients who will need alternative levels of care, such as an intensive care unit (ICU), hospice, and full-time medical care, versus those who are likely to be discharged home. Machine learning (ML) methods may help identify some of those patients early in the admittance process by screening a patient’s electronic health records (EHR) to predict whether or not they may require more hospital resources, helping to allocate resources more effectively.

Big data is currently being used in various applications throughout healthcare facilities, such as identifying high-risk patients in hospitals to better care for patients with a wide array of medical conditions [4–6]. Supervised ML methods such as decision trees and support vector machines (SVM) have been used in assisting or improving upon disease diagnoses and have been employed for other uses within disciplines like biomedical informatics [7, 8]. Machine learning has previously been shown to extract risk factors from EHR that can help predict outcomes [9–11]. One study highlighted the benefit of ML aided by EHR data in identifying patients who were most at risk for posthospitalization venous thromboembolism (VTE) [12].

Numerous ML models have been devised to help identify patients who are at risk of being transferred to an intensive care unit within 7 days of hospitalization and predict mortality in elderly hospitalized patients [3]. These types of models require data gathered throughout a series of tests conducted in the hospital which can take several hours once a sample has been collected. It is important to be able to identify information as early as the triage stage in a hospital environment that can allow the medical staff to better evaluate the resources a patient may require before several tests are completed. This can potentially lead to better allocation of resources and save staff time while allowing them to provide a better level of care for patients [13–17].

The current state of the literature shows ML techniques have been used in computational epidemiology, early detection, diagnosis, and disease progression across various applications and implementations. On review, we identified 19 studies that focused on predicting disease progression and outcomes [18]. Two of those studies focused on the risk stratification of patients in order to allocate medical resources appropriately during the COVID-19 crisis [18, 19]. Yadaw et al. used ML to identify a COVID-19 mortality predictor based on 5 clinical features: age, minimum oxygen saturation during encounter, type of patient encounter, hydroxychloroquine use, and maximum body temperature [20]. Another study created ML models to predict severe pneumonia patients from nonsevere pneumonia patients. Cohorts for these studies were 5,051 and 86 patients respectively [20, 21]. Based on the literature, our study and models contribute by identifying patients at high risk of being admitted to some form of medical care (ICU, hospice, rehab, etc.) versus patients who are likely to be discharged home.

## Methods

### Dataset

The data used for this study were acquired through the University of Florida Health (UF Health) Integrated Data Repository (IDR). The UF Health IDR is an electronic data warehouse that collects and stores information from the UF Health Epic EHR system and other clinical information systems to support research, clinical and operational analysis, and reporting. The study dataset is a deidentified COVID-19 registry that was created from the IDR warehouse and is updated regularly to support COVID-19 related research. The registry includes demographic, medical history, diagnosis, medications, laboratory results, vitals, and hospital utilization information for patients presenting with COVID-19-like symptoms and/or having undergone COVID-19 clinical testing at UF Health systems across Gainesville and Jacksonville since January 1, 2020. The UF Institutional Review Board approved the registry for use in research studies. The data in this study were deidentified to protect sensitive patient information.

This COVID-19 registry is formatted in the Observational Medical Outcomes Partnership (OMOP) Common Data Model. For de-identification/confidentiality purposes, dates are randomly shifted forward or backward with temporality between dummy dates preserved within a given individual. For this study, we used the UF Health COVID-19 registry version 5.0, which included patient data from January 1, 2020 to November 8, 2020 and was processed for this study in October 2021. Within the OMOP-formatted registry, we analyzed data from seven clinical data tables, which were linkable by the person ID variable: person table (contains patient demographics), death table (contains death date collected from EHR and Social Security Death Index), visit occurrence table (contains encounter-level information), condition occurrence table (contains conditions, diagnosis codes, etc.), procedure occurrence table (contains most procedures), drug exposure table (contains all medication orders), and measurement table (contains various labs, vitals, and flowsheet values). S1 Table was derived from these seven tables, outlining the 129 features in the dataset. In Table 1 the p-values for continuous variables were calculated with a Wilcoxon rank-sum test and categorical variable p-values was calculated using the chi-square test.

**Table 1:** Patient characteristics.

Variables	Discharged home (Yes)	Discharged home (No)	P-value
DEMOGRAPHICS			
Age, mean (std)	51.09 (17.72)	63.03 (19.83)	< .0001
Male sex	515 (38.20%)	103 (44.78%)	0.06
Race			0.72
White	633 (46.96%)	108 (46.96%)	
Black	694 (51.48%)	120 (52.17%)	
Other	21 (1.56%)	2 (0.87%)	
Hispanic or Latino	36 (2.67%)	3 (1.30%)	0.35
CLINICAL CHARACTERISTICS			
Heart rate, mean (std)	86.89 (16.32)	87.14 (19.54)	0.92
Respiratory rate, mean (std)	18.73 (4.53)	19.89 (5.33)	0.0015
Body temperature, mean (std)	37.15 (0.68)	37.22 (0.82)	0.41
Body height, mean (std)	169.26 (11.56)	169.36 (10.91)	0.96
Body weight, mean (std)	94.32 (26.20)	89.41 (28.10)	0.0006
COMORBIDITIES (0: Not present, 1: Present)			
Essential hypertension	808 (59.94%)	164 (71.30%)	0.0011
Hyperlipidemia	553 (41.02%)	123 (53.48%)	0.0004
Type 2 diabetes mellitus without complication	389 (28.86%)	91 (39.57%)	0.0011
Chronic pain	492 (36.50)	92 (40.00%)	0.31
Type 2 diabetes mellitus	431 (31.97%)	106 (46.09%)	< .0001
Gastroesophageal reflux disease without esophagitis	443 (32.86%)	80 (34.78%)	0.57
Generalized anxiety disorder	178 (13.20%)	28 (12.17%)	0.67
Low back pain	382 (28.34%)	45 (19.57%)	0.01
Anxiety disorder	285 (21.14%)	45 (19.57%)	0.59
Allergic rhinitis	341 (25.30%)	41 (17.83%)	0.01
Obesity	489 (36.28%)	68 (29.57%)	0.05
Vitamin D deficiency	285 (21.14%)	38 (16.52%)	0.11
Obstructive sleep apnea syndrome	258 (19.14%)	48 (20.87%)	0.54
Insomnia	245 (18.18%)	32 (13.91%)	0.12
Osteoarthritis of knee	205 (15.21%)	35 (15.22%)	0.9970
Morbid obesity	303 (22.48%)	45 (19.57%)	0.32
Hyperglycemia due to type 2 diabetes mellitus	192 (14.24%)	46 (14.24%)	0.02
Atherosclerosis of coronary artery without angina pectoris	165 (12.24%)	61 (26.52%)	< .0001
Hypothyroidism	160 (11.87%)	41 (17.83%)	0.01
Lumbago with sciatica	166 (12.31%)	17 (7.39%)	0.03
Neck pain	209 (15.50%)	33 (14.35%)	0.65
Shoulder joint pain	260 (19.29%)	39 (16.96%)	0.40
Anemia	384 (28.49%)	94 (40.87%)	0.0002
Major depression, single episode	282 (20.92%)	63 (27.39%)	0.03
Chest pain	487 (36.13%)	91 (39.57%)	0.32
Mixed hyperlipidemia	179 (13.28%)	35 (15.22%)	0.43
Acute upper respiratory infection	370 (27.45%)	37 (16.09%)	0.0003
Hypomagnesemia	89 (6.60%)	34 (14.78%)	< .0001
Chronic obstructive lung disease	108 (8.01%)	37 (16.09%)	< .0001
Congestive heart failure	141 (10.46%)	54 (23.48%)	< .0001
Acquired hypothyroidism	102 (7.57%)	19 (8.26%)	0.71
Atrial fibrillation	96 (7.12%)	44 (19.13%)	< .0001
Disorder of phosphorus metabolism	77 (5.71%)	35 (15.22%)	< .0001
High-risk pregnancy	51 (3.78%)	3 (1.30%)	0.06
Nicotine dependence	133 (9.87%)	38 (16.52%)	0.0027
Urinary tract infectious disease	221 (16.39%)	56 (24.35%)	0.0034
Iron deficiency anemia	213 (15.80%)	45 (19.57%)	0.15
Vitamin B deficiency	127 (9.42%)	26 (11.30%)	0.37
Peripheral vascular disease	76 (5.64%)	26 (11.30%)	0.0012
Constipation	224 (16.62%)	46 (20.00%)	0.21
Abdominal pain	299 (22.18%)	47 (20.43%)	0.55
Benign essential hypertension	173 (12.83%)	33 (14.35%)	0.53
Depressive disorder	161 (11.94%)	28 (12.17%)	0.92
Primary malignant neoplasm of female breast	31 (2.30%)	8 (3.48%)	0.29
Hip pain	133 (9.87%)	23 (10.00%)	0.95
Pure hypercholesterolemia	148 (10.98%)	26 (11.30%)	0.88
Gastroesophageal reflux disease	470 (34.87%)	83 (36.09%)	0.72
Chronic pain syndrome	92 (6.82%)	25 (10.87%)	0.03
Diarrhea	223 (16.54%)	39 (16.96%)	0.88
Paroxysmal atrial fibrillation	53 (3.93%)	23 (10.00%)	< .0001
Chronic kidney disease	222 (16.47%)	70 (30.43%)	< .0001
Acute pharyngitis	245 (18.18%)	14 (6.09%)	< .0001
Osteoarthritis	339 (25.15%)	78 (33.91%)	0.01
Chronic systolic heart failure	67 (4.97%)	19 (8.26%)	0.04
Allergic rhinitis due to pollen	135 (10.01%)	12 (5.22%)	0.02
Fatigue	345 (25.59%)	44 (19.13%)	0.04
Backache	221 (16.39%)	39 (16.96%)	0.83
Third trimester pregnancy	54 (4.01%)	2 (0.87%)	0.02
Chronic congestive heart failure	66 (4.90%)	17 (7.39%)	0.12
Moderate recurrent major depression	60 (4.45%)	8 (3.48%)	0.50
End-stage renal disease	56 (4.15%)	13 (5.65%)	0.30
Osteoporosis	67 (4.97%)	20 (8.70%)	0.02
Seizure	59 (4.38%)	20 (8.70%)	0.01
Hypokalemia	140 (10.39%)	62 (26.96%)	< .0001
Asthma	95 (7.05%)	12 (5.22%)	0.31
Joint pain	150 (11.13%)	24 (10.43%)	0.76
Mild intermittent asthma	98 (7.27%)	12 (5.22%)	0.26
Second trimester pregnancy	45 (3.34%)	2 (0.87%)	0.04
Cirrhosis of liver	18 (1.34%)	7 (3.04%)	0.06
Posttraumatic stress disorder	40 (2.97%)	9 (3.91%)	0.41
Gastroparesis syndrome	48 (3.56%)	9 (3.91%)	0.79
Fibromyalgia	47 (3.49%)	7 (3.04%)	0.73
Nutrition deficiency	565 (41.91%)	133 (57.83%)	< .0001
MEDICATIONS (0: Not present, 1: Present)			
ACE	433 (32.12%)	91 (39.57%)	0.03
ARB	255 (18.92%)	54 (23.48%)	0.11
HCQ	29 (2.15%)	11 (4.78%)	0.02
Steroid	814 (60.39%)	150 (65.22%)	0.16

ACE, angiotensin-converting enzyme; ARB, angiotensin receptor blocker; HCQ, hydroxychloroquine.

### Data preprocessing

We identified adult patients aged 18 and older who were tested for COVID-19 within the UF Health system during January 2020 to November 2020. We focused on adult patients in our study as the incidence of positive COVID-19 tests was relatively low among children in the early stages of the pandemic compared to adults [22–24]. Furthermore, the complex disease phenotype associated with COVID-19 in children made it challenging to generalize our findings to this population. For patients with one or more COVID-positive test results, we identified the first date of a positive test result as the index date. We included 1,656 adult patients who had tested positive for COVID-19 in this study cohort. We excluded patients without any COVID-19 test results, patients <18 years old at index date, patients who did not appear in the condition occurrence data table, and those who tested negative for COVID-19, resulting in 1,578 total patients. To generate the main outcome of the study, we grouped 18 discharge codes to a binary variable by categorizing whether the patient was discharged home (a value of 1) or sent to another type of medical care facility (0). Lastly, oversampling of the minority class was used to balance the data for ML models. There were no missing data in the final group of patients for this study.

Of the 129 features in S1 Table, 18 were unique discharge codes to different locations to which the patients were discharged. This was condensed to either discharged home (discharge: 1) or to alternative medical care (discharge: 0) as the primary outcome measured. Features that included sensitive data, such as patient ID or other date-related data, were removed from the final subset as well. Lastly, the feature selection process resulted in 91 features being selected as explanatory variables.

### Outcome

The predicted outcome was whether a patient was discharged home (class 1) or to an alternative source of care (class 0).

### Machine learning algorithm description

To build the classification models, we used 6 ML algorithms (logistic regression, Gaussian Naïve Bayes, k-nearest neighbors, decision trees, random forest classifier, and support vector machine classifier) as prediction-based algorithms, using Python 3.8 and sklearn to build the models [25]. These models were selected based on their ability to identify patterns in classification tasks with a binary outcome. Each of the models was selected based on its strong record of reported outcomes in ML models that have been published using EHR data or other medical data.

Logistic regression (LR) is a classification function that relies on determining the boundary between classes 0 or 1. Given the size of the data and 91 features identified in the data, logistic regression has been shown to find strong associations with an outcome [26], which is the goal of this study. As the size of the dataset expands, the model has a tendency to become more robust in its ability to handle various data patterns, resulting in enhanced classification performance and rendering it one of the frequently employed ML models [27].

K-nearest neighbors (KNN) is an algorithm that classifies objects based on the proximity of the objects in the training set. One of the benefits of this algorithm is that the only adjustable parameter in the model is k which is the number of neighbors that need to be considered and used for an estimation of classification [28, 29]. This allows it to be adaptive to relatively noisy training data and simple to implement with EHR data when determining the class of each patient.

Decision trees (DT) operate by repeatedly splitting the data set which results in a tree-like structure for determining the best criteria to make a classification decision [27]. One of the key benefits of using decision trees is their ability to be easily interpreted based on the rules created during training. When making medical decisions it is important to know what factors impact those decisions, and the tree-like structure can provide the information necessary to better clarify the decision-making process [30–32].

The random forest (RF) method uses a combination of classifiers that builds off multiple decision trees for classification tasks. Each decision tree can provide several leaf nodes along with various depths for each tree; random forest can then combine the calculations to determine optimal results for producing the model. By building models of several different lengths and features, random forest is often found to be insensitive to overfitting [27, 33, 34].

Gaussian Naïve Bayes is an algorithm that assumes all the features are independent of each other and that each class follows a Gaussian distribution. Since the data have both discrete and continuous variables, this type of classifier has been shown to work well in classification tasks if the assumption is not met [35, 36].

Lastly, support vector machine classifier (SVC) is a linear or nonlinear classification model that finds the hyperplane that best separates the classes in the feature space. The model maximizes the margin between the hyperplane and the closest data points, which are the support vectors. An SVC can handle both binary and multiclass classification problems which allows it to be effective in high-dimensional feature spaces [37].

### Interpretable machine learning

Shapley Additive Explanations (SHAP) values using the SHAP library were calculated to identify the individual contributions of each model’s features [38].

### Training, cross-validation, and testing

The data were first randomly divided in an 80/20 split to create a training set for model development and a testing set for model evaluation. The training set was calibrated using Platt’s method and 5-fold-cross-validation and it included 1,262 observations, with 1,083 observations of patients discharged home and 179 observations of patients discharged to an alternative level of care. Hyperparameter tuning was done through grid search with the following parameters for LR parameters c (0.1,1,10), KNN n neighbors (3,5,7), DT max depth (None, 10,20), RF n estimators (100,200,300), and SVC c parameter (0.1, 1, 10). Each model was then evaluated on the 20% test set based on the area under the receiver operating characteristic curve (ROC-AUC) along with accuracy, precision, recall, sensitivity, F1-score, and Brier score.

## Results

### Baseline characteristics

This study cohort included 1,578 patients: 960 (60.8%) female patients with mean (SD) years of age of 51.86 (18.49) years and 618 (39.2%) male patients with mean (SD) years of age of 54.35 (18.48). The patients were randomly divided into two sets (1,262 and 316 respectively): a training set (80%) and a testing set (20%) each comprised of 91 features. Of the 1,578 patients in the cohort, 230 patients were discharged to an alternative level of care and 1,348 were discharged home.

### Comparison and selection of machine learning algorithms

**Fig 1:**
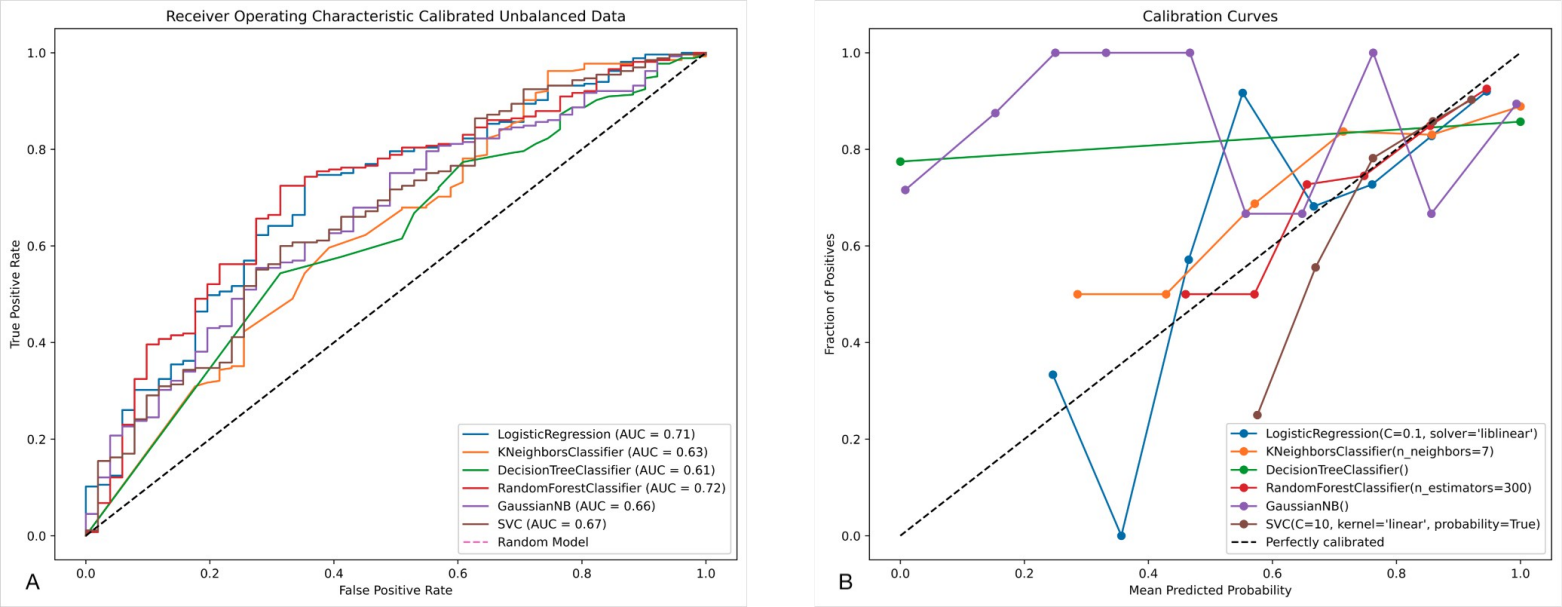
ROC-AUC and ML calibration plot. (A) ROC-AUC, area under the receiver operating characteristic curve. (B) Calibration plot of machine learning models.

Table 2 shows the metrics that were calibrated for each model: AUC, accuracy, specificity, recall, precision, F1 and Brier score. Based on the results, random forest (RF) achieved the highest metrics for AUC (0.72), while logistic regression (LR) achieved the highest accuracy (0.85), specificity (0.10), precision (0.85), and F1 (0.92). As shown in Fig 1B, the RF model was best calibrated to work with this classification problem.

**Table 2:** Algorithm metrics.

Classifier	AUC	Accuracy	Specificity	Recall	Precision	F1	Brier Score
Logistic regression	0.71	**0.85**	**0.10**	0.99	**0.85**	**0.92**	0.13
K-nearest neighbors	0.63	0.84	0.00	**1.00**	0.84	0.91	0.13
Decision tree	0.61	0.84	0.00	**1.00**	0.84	0.91	0.13
Random forest	**0.72**	0.84	0.06	0.98	0.84	0.91	0.13
Gaussian NB	0.66	0.84	0.00	**1.00**	0.84	0.91	0.13
SVC	0.67	0.84	0.04	0.99	0.84	0.91	0.13

AUC, area under the curve; NB, Naïve Bayes; SVC, support vector machine classifier.

### Interpretable machine learning using Shapley values

**Fig 2:**
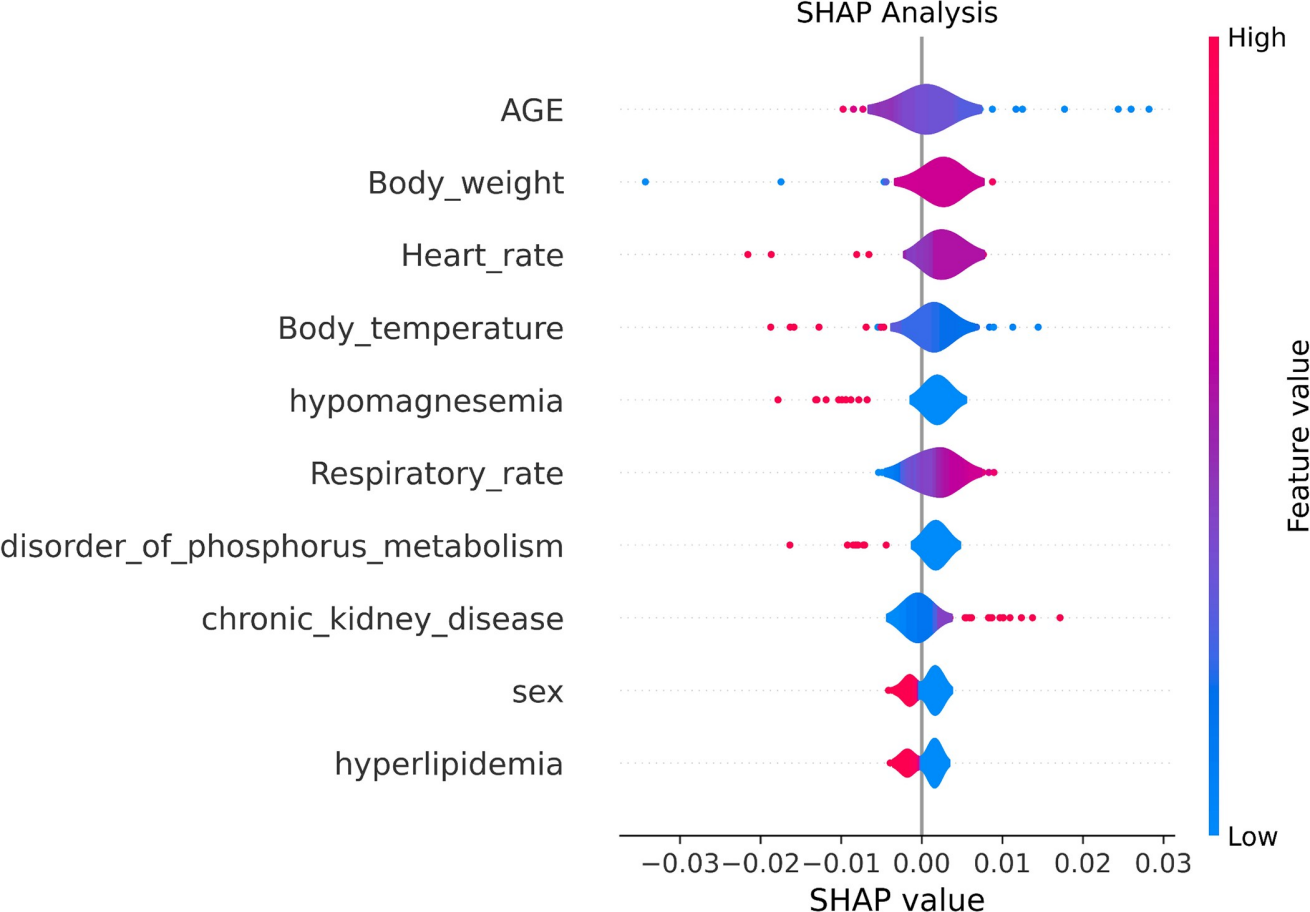
Violin plot Shapley values.

To identify which of the 91 features were important to each model, we calculated SHAP values [38] as seen in Fig 2. SHAP analysis assigns a SHAP value for each feature based upon its impact to the model. The x-position of the dot is determined by the SHAP value of the feature, and as the number of dots increase for each feature the importance increases. Based on the results, the top features identified are age, body weight, heart rate, body temperature, hypomagnesemia, respiratory rate, disorder of phosphorus metabolism, chronic kidney disease, sex, and hyperlipidemia.

## Discussion

The purpose of this study was to establish a ML-based system in which information that is captured during triage would allow medical institutions to identify COVID-19–positive patients who would be discharged home or to an alternative medical facility. In prior studies, several ML models were developed to study identification of individuals at risk of COVID-19 and possible outcomes [19, 39]. Out of the 593 studies identified by Wynants et al., only one used the approach of interpretable ML to better understand the mortality of COVID-19 [40]. Our study utilized an interpretable ML approach to understand patient outcomes based on their EHR. This approach allowed us to better understand the factors that contribute to a patient’s outcome, such as whether they will be discharged home or require an alternative level of care.

The data used in the study had 1,348 patients that were discharged home and 230 which shows an unbalance in classes. Calibration of the models was done by using Platt’s method along with training and validation through 5-fold cross-validation to improve the reliability of the models. Machine learning classifiers can help as early as the triage stage to identify patients who may require additional treatment versus those who are likely to be discharged home, assisting in the effective allocation of medical resources. Among the ML models trained, the random forest algorithm performed the best out of the 6 tested. Based on the 91 features, random forest had an accuracy of 0.84, and a 0.72 ROC-AUC. The top 10 features identified through SHAP analysis were age, body weight, heart rate, body temperature, hypomagnesemia, respiratory rate, disorder of phosphorus metabolism, chronic kidney disease, sex, and hyperlipidemia.

Currently in the United States, when patients arrive in the emergency department they are screened at check-in, and information such as vitals, medical issues, symptoms, and demographics are captured during the triage stage. During triage, medical staff sort patients based on health and need for medical attention. Each patient is then seen by a medical provider who will recommend a course of treatment. Given the rate of vaccine adoption and new strains of COVID-19, the use of ML can assist medical staff with identifying patients who are going to need more medical attention than others as early as the triage stage. SHAP analysis displays the average impact that the top 10 variables have on identifying which patients are more likely to be transferred to alternative care (Class 0). The medical conditions inferred from the model, as seen in Fig 1, are in alignment with existing literature regarding the severity and potential hazards related to COVID-19. Notably, the inclusion of both heart disease [39, 41] and hypomagnesemia [42] are both consistently found in literature. These conditions could provide another set of questions to be asked during triage to help staff identify factors that could help clinicians monitor patients more closely. Despite the advantages that ML provides, the development of a model does not address the integration of the model into clinical practice. Future work must be done within implementation science to assess the impact on clinical workflows and care delivery.

## Limitations

Our study has some limitations, starting with the fact that the data used in this retrospective study were based on UF Health which may not be representative of the general population. Next, we only used an adult population from the records captured at UF Health facilities in Gainesville, FL and Jacksonville, FL, which may have provided an incomplete medical health history if the patient was treated elsewhere. There is still a demographic of individuals as young as newborns to 17-year-old patients who may not have medical conditions that are more commonly found in adult populations. Other information that could be provided in current EHR is the vaccination status of the patients as it was unavailable at the time of this study. Other important health metrics such as pO_2_/FiO_2_ ratios (1PF ratios) that have been observed as markers for acute respiratory failure in patients with COVID-19 were not available in the dataset for this study [43, 44].

Given the low number of patients that were administered to alternative levels of care, the data was imbalanced with a higher number of observations for patients sent home. With imbalanced datasets, it is difficult for machine learning models to have a high specificity score when evaluated. Techniques such as oversampling the minority class in the training set has been shown to improve metrics overall, but in our case, performance was worse. Since this is a retrospective study, we would still need to validate the model in a live clinical environment or external population to optimize the use of ML in a clinical environment and to overcome the data imbalance issue.

## Conclusions

Machine learning algorithms have achieved several good performances in various aspects of medicine, such as identifying patients who are likely to be admitted to ICUs as well as medical diagnostics. Triage is a step in the clinical workflow that would be one of the earliest opportunities to incorporate ML in identifying patients with COVID-19 who may need more medical attention and resources. Our results show that our model can predict whether a patient will be discharged home compared to other treatment in hopes of providing more patients a discharge home rather than an alternative care facility.

## Supporting information

S1 Table COVID-19 data dictionary. Values with a * next to the name are features for data analysis and machine learning models.
(PDF)
