# Supplementary material for: Machine learning-based prediction models for home discharge in patients with COVID-19: Development and evaluation using electronic health records
Source: PLoS One. 2023 Oct 20;18(10):e0292888. doi: 10.1371/journal.pone.0292888 (PMC10588875; doi:10.1371/journal.pone.0292888)
Supplement: S1 Table — Values with a * next to the name are features for data analysis and machine learning models. (PDF) [file pone.0292888.s001.pdf]

S1 Table. COVID-19 data dictionary. Values with a * next to the name are features for data analysis and machine learning models.

Variable	Format/Values	Description
Participant_id	_Integer	Unique patient ID
measurement_date	YYMMDD10.	
race_white*	White=1; Non-white/missing=0	Derived from race ="White"
race_black*	Black=1; Non-black/missing=0	Derived from race ="Black or African American"
race_other*	Other race=1; white/black/missing=0	Derived from other race combined (race="American Indian or Alaska Native" or "Asian" or "Native Hawaiian or Other Pacific Islander")
AGE*	before removing death: 0-99, mean=47; after removing death: 0-99, mean=45.6	
observation_period_start_date	YYMMDD10.	
observation_period_end_date	YYMMDD10.	
death	Death=1 (pt has a death_date); Live=0 (pt doesn't have a death_date)	Derived from death_date
pre_test	Integer 0 - n	days of enrollment before the 1st COVID test date
post_test	Integer 0 - n	days of enrollment after the 1st COVID test date; should be ≥180 days unless dead
covid_test_date	YYMMDD10.	date of the 1st covid +/- test result (index date)
covid_result*	COVID- =0; COVID+ =1	1st COVID +/- test result
visit_inpt*	Yes=1; No=0	Derived from visit_source_value ="INPATIENT"
visit_outpt*	Yes=1; No=0	Derived from visit_source_value="OUTPATIENT"
visit_er*	Yes=1; No=0	Derived from visit_source_value="EMERGENCY"
visit_ambulatory	Yes=1; No=0	Derived from visit_source_value="AMBULATORY SURGERY"
visit_observation	Yes=1; No=0	Derived from visit_source_value="OBSERVATION"
visit_recur_outpt	Yes=1; No=0	Derived from visit_source_value="RECURRING OUTPATIENT"
visit_pend	Yes=1; No=0	Derived from visit_source_value="CM PENDING"
discharge_1	Yes=1; No=0	Derived from discharge_to_source_value="TO HOME"
discharge_2	Yes=1; No=0	Derived from discharge_to_source_value="TO HOMECARE"
discharge_3	Yes=1; No=0	Derived from discharge_to_source_value="EXPIRED NO AUT"
discharge_4	Yes=1; No=0	Derived from discharge_to_source_value="TO SKILLED NURSING"
discharge_5	Yes=1; No=0	Derived from discharge_to_source_value="TO REHAB"
discharge_6	Yes=1; No=0	Derived from discharge_to_source_value="AMA"
discharge_7	Yes=1; No=0	Derived from discharge_to_source_value="TO HOSPICE FACILITY"
discharge_8	Yes=1; No=0	Derived from discharge_to_source_value="EXPIRED AUT"
discharge_9	Yes=1; No=0	Derived from discharge_to_source_value="TO ANOTHER HOSPITAL"
discharge_10	Yes=1; No=0	Derived from discharge_to_source_value="TO HOSPICE HOME"
discharge_11	Yes=1; No=0	Derived from discharge_to_source_value="TO SHANDS PSYCHIATRIC FACILITY"
discharge_12	Yes=1; No=0	Derived from discharge_to_source_value="TO OTHER PSYCHIATRIC FACILITY"
discharge_13	Yes=1; No=0	Derived from discharge_to_source_value="TO LONG TERM CARE HOSPITAL"
discharge_14	Yes=1; No=0	Derived from discharge_to_source_value="TO ALTERNATIVE LEVEL OF CARE"
discharge_15	Yes=1; No=0	Derived from discharge_to_source_value="LWBS"
discharge_16	Yes=1; No=0	Derived from discharge_to_source_value="TO COURT OR LAW ENFORCEMENT"
discharge_17	Yes=1; No=0	Derived from discharge_to_source_value="TO REHAB PLANNED READMIT"
discharge_18	Yes=1; No=0	Derived from other discharge_to_source_value <0.1% frequencies combined (see characteristics tab)

visit_start_date	YYMMDD10.	
visit_end_date	YYMMDD10.	
CONDITION_1*	Yes=1; No=0	Essential hypertension
CONDITION_2*	Yes=1; No=0	Hyperlipidemia
CONDITION_3*	Yes=1; No=0	Type 2 diabetes mellitus without complication
CONDITION_4*	Yes=1; No=0	Chronic pain
CONDITION_5*	Yes=1; No=0	Type 2 diabetes mellitus
CONDITION_6*	Yes=1; No=0	Gastroesophageal reflux disease without esophagitis
CONDITION_7*	Yes=1; No=0	Generalized anxiety disorder
CONDITION_8*	Yes=1; No=0	Low back pain
CONDITION_9*	Yes=1; No=0	Anxiety disorder
CONDITION_10*	Yes=1; No=0	Allergic rhinitis
CONDITION_11*	Yes=1; No=0	Obesity
CONDITION_12*	Yes=1; No=0	Vitamin D deficiency
CONDITION_13*	Yes=1; No=0	Obstructive sleep apnea syndrome
CONDITION_14*	Yes=1; No=0	Insomnia
CONDITION_15*	Yes=1; No=0	Osteoarthritis of knee
CONDITION_16*	Yes=1; No=0	Morbid obesity
CONDITION_17*	Yes=1; No=0	Hyperglycemia due to type 2 diabetes mellitus
CONDITION_18*	Yes=1; No=0	Atherosclerosis of coronary artery without angina pectoris
CONDITION_19*	Yes=1; No=0	Hypothyroidism
CONDITION_20*	Yes=1; No=0	Lumbago with sciatica
CONDITION_21*	Yes=1; No=0	Neck pain
CONDITION_22*	Yes=1; No=0	Shoulder joint pain
CONDITION_23*	Yes=1; No=0	Anemia
CONDITION_24*	Yes=1; No=0	Major depression, single episode
CONDITION_25*	Yes=1; No=0	Chest pain
CONDITION_26*	Yes=1; No=0	Mixed hyperlipidemia
CONDITION_27*	Yes=1; No=0	Acute upper respiratory infection
CONDITION_28*	Yes=1; No=0	Hypomagnesemia
CONDITION_29*	Yes=1; No=0	Chronic obstructive lung disease
CONDITION_30*	Yes=1; No=0	Congestive heart failure
CONDITION_31*	Yes=1; No=0	Acquired hypothyroidism
CONDITION_32*	Yes=1; No=0	Atrial fibrillation
CONDITION_33*	Yes=1; No=0	Disorder of phosphorus metabolism
CONDITION_34*	Yes=1; No=0	High risk pregnancy
CONDITION_35*	Yes=1; No=0	Nicotine dependence
CONDITION_36*	Yes=1; No=0	Urinary tract infectious disease
CONDITION_37*	Yes=1; No=0	Iron deficiency anemia
CONDITION_38*	Yes=1; No=0	Vitamin B deficiency
CONDITION_39*	Yes=1; No=0	Peripheral vascular disease
CONDITION_40*	Yes=1; No=0	Constipation
CONDITION_41*	Yes=1; No=0	Abdominal pain
CONDITION_42*	Yes=1; No=0	Benign essential hypertension
CONDITION_43*	Yes=1; No=0	Depressive disorder
CONDITION_44*	Yes=1; No=0	Primary malignant neoplasm of female breast
CONDITION_45*	Yes=1; No=0	Hip pain
CONDITION_46*	Yes=1; No=0	Pure hypercholesterolemia
CONDITION_47*	Yes=1; No=0	Gastroesophageal reflux disease
CONDITION_48*	Yes=1; No=0	Chronic pain syndrome
CONDITION_49*	Yes=1; No=0	Diarrhea
CONDITION_50*	Yes=1; No=0	Paroxysmal atrial fibrillation
CONDITION_51*	Yes=1; No=0	Chronic kidney disease
CONDITION_52*	Yes=1; No=0	Acute pharyngitis
CONDITION_53*	Yes=1; No=0	Osteoarthritis
CONDITION_54*	Yes=1; No=0	Chronic systolic heart failure
CONDITION_55*	Yes=1; No=0	Allergic rhinitis due to pollen
CONDITION_56*	Yes=1; No=0	Fatigue
CONDITION_57*	Yes=1; No=0	Backache
CONDITION_58*	Yes=1; No=0	Third trimester pregnancy
CONDITION_59*	Yes=1; No=0	Chronic congestive heart failure
CONDITION_60*	Yes=1; No=0	Moderate recurrent major depression
CONDITION_61*	Yes=1; No=0	End-stage renal disease
CONDITION_62*	Yes=1; No=0	Osteoporosis
CONDITION_63*	Yes=1; No=0	Seizure

CONDITION_64*	Yes=1; No=0	Hypokalemia
CONDITION_65*	Yes=1; No=0	Attention deficit hyperactivity disorder, combined type
CONDITION_66*	Yes=1; No=0	Asthma
CONDITION_67*	Yes=1; No=0	Joint pain
CONDITION_68*	Yes=1; No=0	Mild intermittent asthma
CONDITION_69*	Yes=1; No=0	Second trimester pregnancy
CONDITION_70*	Yes=1; No=0	Cirrhosis of liver
CONDITION_71*	Yes=1; No=0	Posttraumatic stress disorder
CONDITION_72*	Yes=1; No=0	Gastroparesis syndrome
CONDITION_73*	Yes=1; No=0	Fibromyalgia
Sex*	Female=0, Male=1	Derived from gender
Ethnic*\	Hispanic or Latino=1; Not Hispanic or Latino=0	Derived from ethnicity
ACE*		Use of ACE Inhibitors
ARB*		Use of ARBs
HCQ*		Use of Hydroxychloroquine
STEROID*		Use of Steroids
Body_temperature*		
Heart_rate*		
Body_weight*		
Respiratory_rate*		
Body_height*		
nutrition_deficiency	if any of (Hypokalemia, Vitamin B deficiency, Iron deficiency anemia, Hypomagnesemia,Vitamin D deficiency) =1 then equal to 1, else equal to 0	
